# Authors' reply

**Published:** 2010

**Authors:** Vipul L Arora, Kim R Usha, Hadi M Khazei, Shivayogi Kusagur

**Affiliations:** Orbit, Oculoplasty and Oncology Clinic, Aravind Eye Hospital and Postgraduate Institute of Ophthalmology, Madurai, Tamil Nadu, India

Dear Editor,

The authors wish to thank Gonzalez-Granado[1] for his interest in our article[2] and for his comments.

1. Regarding the etiology of the disease, we have mentioned about the chromosome 4q.[3,4] Further details of genetic mutations associated with this syndrome were not within the scope of this case report. The authors appreciate that this point was brought into focus.
2. Patient was prescribed azithromycin after discussing it with the dermatologist. The patient had high serum IgE of 7375.2 IU/ml, there was a risk of further deterioration with sulpha drugs (cotrimoxazole) or pencillin group (cephalosporins), as these drugs are known to cause hypersensitivity reaction, which is an IgE-mediated response. Moreover, azithromycin has good activity against the staphylococcus group.[5]
3. We agree that there is no other case report which suggests the association of Job's syndrome with retinal detachment. Therefore, we have mentioned in our case report that *“we speculate that the etiology and pathogenesis of retinal detachment in case of Job's syndrome closely resembles with that of atopic dermatitis, which is an IgE-mediated hypersensitivity reaction”*. This suggests that more studies are needed to prove this hypothesis.
